# Ecolabeled seafood and sustainable consumption in the Canadian context: issues and insights from a survey of seafood consumers

**DOI:** 10.1007/s40152-021-00245-y

**Published:** 2021-09-27

**Authors:** Anthony Winson, Jin Young Choi, Devan Hunter, Chantelle Ramsundar

**Affiliations:** 1 Department of Sociology and Anthropology, University of Guelph, Guelph, ON N1G2W1 Canada; 2 Department of Sociology, Sam Houston State University, Huntsville, TX USA; 3 Department of Sociology and Anthropology, University of Guelph, Guelph, Canada; 4 School of Public Health and Health Systems, University of Waterloo, Waterloo, Canada

**Keywords:** Canada, Ecolabel, Seafood, Sustainable consumption, Survey

## Abstract

The concept of sustainable consumption is a much debated practice that has been seen as an outcome of the emergence of *ecological citizenship*—a concept that brings together the citizen and the environment in a framework that is underlined by social justice considerations and incorporates a vision of citizenship that involves both the private sphere and the public sphere of human activity. This study examines Canadian consumer awareness and uptake of certified sustainable seafood. We introduce the concepts *ecological citizenship* and *sustainable consumption* as a way of framing our research. Seafood ecolabels may be a valuable tool in translating general environmental concern about the marine environment into more sustainable fisheries practices. We conducted an on-site consumer survey in the Greater Toronto Area and a nearby city. Our findings showed that in contrast to high levels of awareness of the importance of the marine environment and the sustainability of seafood, consumers had a limited understanding about the meaning of sustainability in the case of seafood, and little knowledge about actual ecolabels found in the Canadian marketplace. Attitudes towards the marine environment and sustainable seafood, understanding of the meaning of seafood sustainability, and purchasing behaviors of sustainable seafood were significantly different by some socio-demographic characteristics. Positive attitudes towards the marine environment and sustainable seafood and better understanding of seafood sustainability were significantly associated with the increased purchasing of ecolabeled seafood. Lack of understanding of ecolabels, limited information about product sustainability, and lack of in-store guidance were identified as key barriers to purchasing ecolabeled seafood products.

## Introduction

In a world characterized by rapid environmental degradation and species decline and extinctions, the practice of sustainable consumption has been advocated by many as a viable solution, or at least part of the answer, to reversing current trends. With respect to the gamut of pressing environmental issues we face today, the critical state of *marine* environments around the globe is particularly salient. There is growing consensus that industrial fishing has impacted fish stocks around the world with dramatic declines in large predatory fish populations (Myers and Worm 2003; Worm et al. 2006) where overfishing and the bycatch from fishing operations have seriously impacted such keystone species as sharks and rays that are crucial for marine ecosystem stability and functioning (Birkmanis et al. 2020).

The popularization among expanding middle-class consumers of what were once elite cultural practices, such as serving shark fin soup at weddings and other special occasions, has been particularly devastating for these marine apex predator populations (Oceans Asia 2021). The failure to harvest many fish stocks sustainably and the complex difficulties in crafting effective policies regarding fisheries (Jentoft and Cheunpagdee 2009) have led to the stagnation of global catches despite increased fishing effort (Longo et al. 2015; Orlowski 2019; Sinclair 1997; Sundar 2016). The implementation of management measures *has* achieved some success in sustaining viable stocks of such important species as tuna (ISSF 2020; FAO 2020:8)^1^and significant progress has been made bringing large supermarket chain store, food service, and fast food giants on board supporting sustainable seafood. Nevertheless, still by 2017, the Food and Agriculture Organization (FAO 2020:7) reported that 34.2% of fish stocks were being fished at biologically unsustainable levels. Moreover, in addition to ecological impacts, fisheries decline affects global food supply and food security, food safety, North–South dimensions of food trade, and coastal livelihoods (Ponte 2012; Vandergeest and Unno 2012; Vandergeest et al. 2015; Sundar 2016).

Within this context of rapid depletion of numerous marine species we take for food, the issue of harvesting and consuming seafood in a more sustainable manner has gained prominence in recent times, particularly in terms of private sector environmental NGO initiatives and to some extent state policies. Sovereign nations have a long history of fisheries regulation, but this regulatory power had limited effectiveness in dealing with the decline of fisheries. Responding to this failure, the World Wildlife Fund and Unilever in 1997 established the Marine Stewardship Council (MSC) which built on one of the few examples of inter-governmental governance initiatives that provided some standards and principles for the fishing industry—the Food and Agriculture Organization’s (FAO) Code of Conduct for Responsible Fisheries—and launched an ecolabeling scheme to support sustainable fisheries management practices. To receive the MSC ecolabel, seafood harvested must meet three basic principles: fishing must allow for the fish stock to be sustainable into the future; the fishing activity must minimize environmental impact; and the fishing operations must be well managed. MSC certified product has a “chain of custody standard” where product is tracked along the entire supply chain. While there was some initial state resistance to the MSC’s non-state management regime, overtime important state support in the developed world has been provided to it. Subsequently, other private seafood ecolabeling schemes have emerged, although typically most claim compliance to the FAO guidelines (Gulbrandsen 2012). With the existence of numerous seafood ecolabels today in many retail food environments, it is important to know more about the level of consumer knowledge and engagement with them, any barriers to the uptake of ecolabeled seafood, and the mechanisms and initiatives that might be useful to increase this uptake and thereby help ensure fish stocks survive and thrive in the future.

The research reported here examines consumers’ awareness of the importance of the marine environment and their understanding and engagement with sustainably sourced marine species we take for food by employing a consumer survey conducted on-site in central Canada. Our research has relevance for the debates on the concept of sustainable consumption, the efficacy of consumers as drivers of change, and the broader theory on ecological citizenship. It also provides some unique insights into the Canadian context where survey research on these issues has been largely absent.

Canada is a major producer of seafood with just over one million tons of seafood harvested annually, of which 83% of this from wild marine fisheries. About 85% of the value of Canada’s captured fish and aquaculture production is exported, largely to the USA, with the principal exports being lobster, snow/queen crab, shrimp, and farmed salmon (Govender et al.^2016^:11). It also imports over one half million tons of seafood, with the top imports being fish meal and fish oil products, shrimp, herring, lobster, farmed salmon, and tuna (Govender et al. 2016:19). Notably, in recent years, the Canadian fishing industry has made some progress in transitioning to sustainable practices with respect to seafood harvested *in* Canadian waters, with 66% of wild marine and freshwater fisheries production certified or in assessment by the Marine Stewardship Council as of 2016. Similar progress does not characterize seafood imported into the country, however. About 30% of imports, mostly fish meal and fish fats, have no species information linked to them, and therefore, it has not been possible to ascertain their sustainability (Govender et al. 2016:14,17, 22).

In this paper, we consider first the concept of ecological citizenship and its relationship to the practice of sustainable consumption. We provide a brief background to the development of seafood ecolabels and some basic details of those ecolabels found in the Canadian marketplace.

We then present our findings from our survey of seafood consumers and discuss them in light of the global literature on sustainable seafood and seafood ecolabels and consider the key barriers consumers reported to expanding their acceptance of sustainable seafood. We conclude with some remarks about the relationship of such private governance mechanisms as seafood ecolabels to state policies in the seafood sector and the role they each play in advancing sustainable seafood.

## Ecological citizenship and sustainable consumption

The recalcitrance of many governments in the prevailing neo-liberal political economy to effectively implement environmental protection has meant that for some, the prospect of positive action on the environment is increasingly vested in the pro-active citizen/consumer. In this regard, the broad concept of *ecological citizenship* advanced by Dobson (2003) encompasses a theory of change towards sustainable lifestyles and living standards (Wolf et al. 2009:504). Dobson (2006) also argues the concept “deals in the currency of political relations founded in justice….”. Ecological citizenship emphasizes a republican version of citizenship foregrounding citizen responsibilities and duties to act for the common good over citizen rights that are more central to a liberal citizenship model, and it involves action in both the private and public sphere (Dobson 2007:280). Ecological citizenship encompasses norms and practices that together promote the reduction of one’s “ecological footprint”—the ecological space each of us take up through resource use and the burden we place on the earth’s carrying capacity (see Wackernagel and Rees 1996). Moreover, in foregrounding the choices and actions of citizen/consumers, it challenges us to know more about the attitudes and knowledge that may enable more ecologically friendly consumption practices. As Seyfang (2005:291) argues, “the challenge is to find the mechanisms and initiatives which enable and encourage people to act as ecological citizens, in other words, to reduce their ecological footprints.” The institutionalization of these norms and practices via a variety of state and private governance arrangements play an increasingly important role in encouraging consumption in an environmentally responsible manner. This brings us to the concept of *sustainable consumption* with its focus on citizen/consumers who incorporate social and environmental concerns in their purchasing decisions and thereby propel market transformation through pressure on upstream producers and suppliers of all manner of goods and services.

As a mainstream market-based approach to achieving a more sustainable economy, sustainable consumption in the form of such mechanisms as ecolabel certification regimes *does* have some significant benefits that should not be dismissed out of hand, if only because it is widely prevalent in capitalist societies and because it has a good deal of political acceptability today and therefore has a better chance of receiving substantial support from both the private sector and government (Seyfang 2005:298). As Seyfang has effectively argued:

The approach does appear to achieve significant benefits in terms of raising awareness of the social and environmental impacts of behavior, and encouraging individuals to think about these and reflect upon the difference they can make through their consumption patterns. It may be that this process is the first step on a journey towards greater education and activity concerning ecological citizenship, and there is certainly scope for strengthening the improvements which mainstream sustainable consumption can make – namely, through government regulation, addressing market failures, and supporting firms in transforming their activities. (2005:296)

There are a number of issues related to this concept, not the least of which concerns the factors that might motivate such behavior, and the barriers to achieving it, matters we return to. As a mechanism for achieving environmental progress, sustainable consumption has also been the subject of criticism. This market-based approach is argued to lead to the further neo-liberalization of society and environmentalism with its focus on individual responsibility for the environment (Konefal 2012:337). Moreover, it has been argued that people may lack good information to consume sustainably, or may lack the opportunity to do so due to the unaffordability and/or unavailability of ethical products (Seyfang 2005:295–296). Finally, it has been argued that the focus on individual consumption decisions means “there is little room to ponder institutions, the nature and exercise of political power, or ways of collectively changing the distribution of power and influence in society” (Maniates 2002:45).

With all these criticisms, does this market-based approach to sustainable consumption have any credibility as a means to address the momentous environmental concerns we face today? While many of these criticisms have a certain amount of validity, sustainable consumption practices may be a useful “stepping stone” towards embracing ecological citizenship in a more meaningful way. Some argue that market-based consumer-oriented programs for marine conservation such as seafood ecolabels may be crucial to translating general environmental concern about the marine environment into more sustainable fisheries practices (e.g., see Barclay and Miller, 2018; Swartz et al., 2017; Wessells et al., 1999). This is particularly the case in the absence of strong governmental efforts at marine conservation, which is the situation in many jurisdictions. Ultimately consumer engagement with sustainably sourced seafood is important if ecolabel certification programs in this industry are to expand and make an appreciable impact in preserving in future marine life we take for food (Jonell et al. 2016). A better understanding of consumer engagement with seafood eco-certification programs is therefore of some importance. With this in mind, we consider some findings of a survey of Canadian seafood consumers. Before doing so, it will be valuable to provide some context.

## Sustainable seafood and seafood certification

Over the last few decades, private and governmental regulatory regimes, standards, and private certification programs that evaluate, rank, and publicize the sustainability of seafood have emerged in Europe and North America (Gulbrandsen 2009; Foley 2012; Ponte 2012; WWF, 2009) as a means to encourage environmentally responsible behavior (Osbaldiston and Shelton 2003) and provide incentives for seafood harvesters to change fishing practices (Arnold and Fuller 2017: 3). It is notable that a growing volume of seafood certified and labeled as “sustainable” has entered North American and European marketplaces since the late 1990s (Ponte 2012). Sustainable seafood consumption is an issue that has also gained increasing prominence in public and social media (Silver and Hawkins 2017).

Seafood certification programs and seafood ecolabels have created new opportunities and challenges along the supply chain from fishers and fishing communities through to end consumers (Foley 2012; Ponte 2012; Hallstein and Villas-Boas 2013; Foley and McCay 2014). Typically funded by private philanthropic foundations, these programs were developed to stand in for what was perceived to be ineffective state governance, or by private firms wishing to differentiate their products by highlighting purported higher standards in harvesting seafood (Roheim et al. 2018:392).

An ecolabel has been described as “a mark, a logo, a label or a product endorsement affixed to a seafood product at the point of sale that implies to a purchaser that the product has been produced through ecologically sustainable procedures, and is from a source that is well managed” (Ward and Phillips 2008:2). Ecolabeling is usually associated with some form of assessment process certifying the product meets a program’s sustainability standard. As Ward and Phillips (2008:3) argue, ecolabels were developed “to create ‘market-pull’ through differential appeal to consumers who are sensitive to the impact on sustainability inferred by the product endorsement of the ecolabel.” Three basic types of ecolabels have been identified (Deere, 1999): (1) first-party labeling schemes typically established by producers or resellers based on their own standards; (2) second-party labeling schemes usually established by industry associations and based on their own certification standards; and (3) third-party labeling schemes that are created by organizations external to the industry and which thereby have a perceived degree of independence in the verification of products according to the standards the particular scheme sets. This last type of ecolabeling scheme is usually considered the most robust of the three types (Ward and Phillips 2008:3).

Seafood ecolabels emerged in the 1970s with the *Dolphin Safe* ecolabel in an effort to curb the massive killing of these mammals that occurred as a result of practices in the yellowfin tuna industry in the tropical Eastern Pacific Ocean. For many years, this was managed under the voluntary International Dolphin Conservation Program with limited success, but in 1999 a more robust legally binding multilateral treaty—the Agreement on the International Dolphin Conservation Program—came into effect to better protect dolphins that includes a tracking and verification system (WWF International, 2009:37). It should be noted that this ecolabel was and is oriented towards protecting marine mammals, and not at conserving tuna stocks, which is an increasingly important issue that is addressed by an ecolabel indicating less destructive fishing methods, such as “pole and line” fishing.

Another major ecolabel program that took shape in the 1990s was that of the *Marine Stewardship Council* (*MSC*). With a focus on the wild capture fishery, MSC is a global organization established by a partnership between the World Wildlife Fund and Unilever in 1997. Fisheries that receive the MSC ecolabel must meet three basic principles: fishing must allow for the fish stock to be sustainable into the future; the fishing activity must minimize environmental impact; and the fishing operations must be well managed. These principles are operationalized by numerous performance indicators that form a fisheries standard that dictates the process whereby a fishery is certified. The MSC certification regime involves a “chain of custody standard” whereby certified fish are tracked along the supply chain from harvesting to final sale (MSC 2021). Since its establishment, the MSC has seen exponential growth and it has established numerous partnerships with retailers, suppliers, restaurant chains, and food service companies (WWF 2009:42). The organization reported in 2019–2020 that 17.4% of the global wild marine catch was certified to the MSC Standard (MSC 2020).

Other seafood ecolabels worthy of note include *Friend of the Sea*, a seafood ecolabel founded by Dr. Paolo Bray of the Earth Island Institute that predated all of the other seafood ecolabel initiatives and had an initial focus on protecting dolphin species by advocating changes in tuna fishing practices. Its focus has broadened to promote sustainable practices in both the wild caught and farmed seafood sectors (WWF International 2009:39). Another well-known ecolabel is *Responsible Fishing Scheme* developed in the UK by the Seafood Industry Authority. It provides an audited assessment of individual vessels with the goal of encouraging operators to demonstrate a commitment to the responsible sourcing of seafood (WWF International 2009:49).

With respect to aquaculture production and in response to the impact of aquaculture operations on other marine species and marine environments, efforts have been made to incentivize aquaculture operations to pursue more sustainable practices. One initiative has been that of the World Wildlife Fund, together with the Dutch Sustainable Trade Initiative, with the formation of the *Aquaculture Stewardship Council (ASC)* in 2010. This ecolabel now covers a variety of species produced via aquaculture (Schiller et al. 2016:95–6). In addition to the ASC, the Global Aquaculture Alliance had previously developed their *Best Aquaculture Practices (BAP)* ecolabel, which is based on a points system whereby points are awarded according to compliance with standards of environmental and social responsibility, food safety, animal health, and traceability. Facilities must meet a minimum number of points to be awarded *BAP* certification (Schiller et al. 2016:96).

In the Canadian context, the rise of seafood ecolabels, including the globally oriented *Marine Stewardship Council*, has been linked to the disastrous collapse of the once prolific cod fishery on the East Coast’s Grand Banks in the early 1990s (Schiller et al. 2016:93). In the absence of effective government action, a few environmental NGOs fostered the development of seafood certification programs to put pressure on food retailers, restaurants, and ultimately consumers to favor sustainably harvested seafood (Bennett 2018). This pressure has helped convince the highly concentrated supermarket chain store sector (Winson 1993) to support sustainably sourced seafood and with their considerable market power they have influenced the entire seafood supply chain.

Two specifically Canadian ecolabel initiatives are worthy of note. *Ocean Wise* was an initiative of the Vancouver Aquarium Marine Science Centre in partnership with Chef Robert Clark. It is a recommendation program that collaborates with the Monterey Bay Aquarium’s Seafood Watch program to provide guidance on sustainable wild caught and farmed seafood to the restaurants, suppliers and retailers it partners with. It lacks resources to conduct audits of its partners, however, to ensure the correct labeling and sourcing of products sold (Schiller et al. 2016: 97–98). *This Fish* is another Canadian ecolabel worthy of mention for its role in providing a traceability program that makes the seafood supply chain more transparent. Fishers upload data on provenance of the fish caught and harvesting methods used which can be accessed by consumers, retailers, and chefs to trace their seafood purchases. This program has expanded internationally as well. While it does not ensure marine species are harvested sustainably, it does promote seafood supply chain transparency and enhanced consumer awareness (Schiller et al. 2016:98).

The seafood ecolabels programs have provided opportunities for more ecologically friendly harvesting methods and more exposure of the general public to marine environmental issues. In the meantime, there have been challenges to the implementation of these programs and debates over their effectiveness (see, e.g., Bundy et al. 2008; Jacquet et al., 2010; Froese and Proelss 2012; Guiterrez et al. 2012; Marine Stewardship Council 2017). This is not surprising given the variety of standards of sustainability that exist and the issues around the compliance assessment process that is typically associated with ecolabeled seafood. In the Canadian context, for example, there have been disagreements among environmental NGOs around the sustainability status of such key species as British Columbia farmed salmon (Bennett 2018).

While ecolabeled seafood has now become a significant part of the food retail environment, it must be noted that numerous studies conducted in several countries have indicated that consumers have limited understanding about marine resources and seafood sustainability issues and knowledge of the meaning of ecolabels (Wessells et al. 1999; Jonell et al. 2016; Pieniak et al. 2013). It is to such issues that we now turn.

## Consumer surveys and sustainable seafood

A substantial number of consumer-oriented surveys focused on sustainably sourced seafood and ecolabel certification programs have been conducted over the years in multiple national contexts. Most of these surveys have been conducted in the USA and European countries, often undertaken in specific metropolitan areas. In an early survey, Wessells et al. (1999) found a low awareness among consumers around the status of fish stocks and argued consumer education as a necessary ingredient to progress in this area. In the analysis of a European survey conducted in seven countries, Pieniak et al. (2013) found that knowledge of the fishing industry was generally low, but that there were significant variations by country. A survey conducted in Stockholm, Sweden (Jonell et al. 2016), showed that respondents had limited knowledge of seafood production systems and the environmental impacts of fish farming and aquaculture more generally. This finding concurs with other studies in Belgium (Verbeke et al., 2007) and even in Japan with the highest per capita seafood consumption in the world (Uchida et al. 2014). By contrast, a recent survey in the US coastal state of Maine (McClenachan et al. 2016) indicated slightly over half of respondents had a good understanding of what was “ecologically sustainable” and what this should mean with regards to ecolabeled seafood.

Recognition and understanding of seafood ecolabels only been examined in a few studies. In Sweden, recognition of seafood ecolabels was found to be low to moderate, with the exception of a specifically Swedish ecolabel, and understanding of non-Swedish ecolabels was low (Jonell et al. 2016). In a small comparative study of one city in the USA and one in the UK, there was also a low level of recognition of seafood ecolabels other than the Dolphin Safe and Organic labels (Guttierez and Thornton, 2014). The Marine Stewardship Council’s 2018a, b survey of 21 countries that focused on its own ecolabel showed that recognition of its label was highest in Switzerland, Germany, and Austria (65% or more), while less than 25% and 21% of respondents recognized the label in the U.S. and Canada, respectively (MSC 2018a, b).

An early study by Johnston et al. (2001) found some heterogeneity across countries with regard to the role of demographic characteristics, with females in Norway but not the USA more likely to select ecolabeled seafood, while high-income consumers were more likely to favor ecolabeled seafood in Norway but not in the USA. More recent studies have found more consistency in terms of what demographic indicators are more likely to predict favorable attitudes and behavior regarding sustainable seafood. In a study conducted in China, Wang and Somogyi (2019) found that a highly positive attitude towards purchasing sustainable seafood was found among those with a high income and a high occupation level (management employees) compared to those with a low monthly income and in lower or medium level occupations. A study of urban consumers in China by Xu et al. (2012) found respondents with a higher education level were somewhat more likely to pay a price premium for ecolabeled seafood. Salladarré et al., 2010:15) found that pro-ecolabel consumers in France were typically well-educated, younger, and urban. Occupationally, consumers who were classified as professionals were more favorable to seafood ecolabels than manual workers and self-employed consumers. Brecard et al. (2009) had similar results from a European survey, with ecolabeled seafood favored more by young, female, and well-educated consumers and consumers who had “intellectual professions,” while “farmers and workers” were less favorable. The study by Perez-Ramirez et al. (2015) in Mexico also noted stronger support for ecolabeled seafood among consumers with higher education and income, while a study in Korea also found high-income earners more likely to favor ecolabeled seafood (Kim and Lee 2018:9). Knowledge about seafood sustainability issues may well be significant because it has been shown to be positively correlated to willingness to purchase sustainable seafood in a number of studies (Lawley et al., 2019; Salladarré et al. 2010). More specifically, recognition and stated understanding of seafood ecolabels has been shown to be positively associated with purchasing of these ecolabels (Jonell et al. 2016:12).

What, then, of the Canadian consumer and his/her awareness and buy-in regarding ecolabeled seafood products? Little research has been conducted in this area. One study employing focus groups did examine whether consumers were willing to purchase and eat seafood produced by more sustainable multi-trophic aquaculture systems (Barrington, 2010). One other study reported on the perceptions of six stakeholder groups towards ecolabeled aquaculture in the province of Nova Scotia (Weitzman and Bailey 2018). In an effort to fill this literature gap, we conducted a survey with a sample of seafood consumers of the Greater Toronto Area and a nearby city in Canada. This survey was the first of its kind in Canada.

## Methods

This study employs an on-site, self-administered survey design. We obtained our sample of adult seafood consumers in two stages. First, we selected six study sites—five supermarkets and a major inner city farmers’ market—in the Greater Toronto Area and a medium size city approximately 1 h to the west of the Greater Toronto Area. The six sites were purposively selected to maximize variation in terms of consumers’ income and ethnicity, as well as a diversity of retail venues (e.g., standard supermarket, discount supermarket, farmers’ market). Permission to survey consumers in each retail location was obtained from the management of the retail organizations before the survey was conducted. A convenience sample of grocery shoppers was taken from the purposively selected six sites. The trained project assistants under the direct on-site supervision of the principal investigator (PI) asked adult shoppers in these sites to participate in the survey during the Spring and Summer of 2018. Those who were younger than 19 years old and those who did not consume seafood were excluded from the survey by utilizing two filtering questions before beginning the survey. The survey procedure followed the protocol approved by the PI’s university research ethics board.

A total of 358 consumers completed the survey. Despite employing a nonprobability sampling design, the ethnic makeup of the sample has close proximity to the Ontario population. Respondents’ household income was somewhat higher than those of the Ontario population in the Canadian census. For example, about 41% of the respondents reported household income above $100 k compared to the Ontario census figure of 35%. Females were slightly over-represented in our sample (57% females vs. 43% males). The sample characteristics are presented in Table 1.

**Table 1:** Sample characteristics

Variables		Percent
Gender^a^		
	Male	43.1
	Female	56.9
Age^b^		
	18–34	28.1
	35–64	56.0
	65 and older	15.9
Ethnicity^c^		
	European and North American	66.9
	Other	33.1
Education^d^		
	Less and equal to high school	15.6
	College, CEGEP, non-bachelor certificate/diploma	26.8
	University	35.8
	Above bachelor or graduate degree	21.8
Household income^e^		
	Under $40,000	16.4
	$40,000 to $99,999	43.0
	$100,000 and over	40.6
Children in the household^f^		
	Yes	34.6
	No	65.4

*n* is different for each variable due to missing cases: ^a^*n* = 357, ^b^*n* = 352, ^c^*n* = 354, ^d^*n* = 358, ^e^*n* = 342 ^f^*n* = 358

A structured survey questionnaire was used. The survey instrument was developed from several existing consumer studies related to recognition and purchasing behavior of ecolabeled seafood (Wessells et al., 1999; Guiterrez and Thornton 2014; Jonell et al. 2016). This allowed us to compare the data with studies of other countries. We also included additional questions that emerged from the investigators’ specific research interests within the Canadian context. The questionnaire was finalized after a pilot test and several revisions. The survey questionnaire was designed to capture information related to respondents’ seafood consumption. In this study, we focused on the following four areas: (1) awareness of the importance of protecting the marine environment and ensuring the sustainability of seafood; (2) understanding the meaning of seafood “sustainability”; (3) awareness of sustainable certification or ecolabels; (4) purchasing seafood with sustainable certification or ecolabel; and (5) perceived barriers to increasing uptake of sustainably sourced seafood. After entering the survey data and data cleaning, descriptive statistics and bivariate analyses (i.e., independent samples *t*-tests, ANOVA, and chi-square tests) were performed for data analysis using SPSS version 22.

## Results

### Awareness of marine environment and sustainability of seafood

Table 2 presents consumers’ attitude toward the marine environment and sustainability of seafood by socio-demographic characteristics. The consumers’ attitude was measured by three items asking respondents’ perceived importance of (a) protecting the ocean environment; (b) protection of marine species we take for food; and (c) harvesting seafood in a sustainable manner. A 5-point scale was used to capture the perceived importance (1 with “not important at all” and 5 with “extremely important”). These three items were merged and a mean value was calculated as a composite construct score (Cronbach’s alpha = 0.927).

**Table 2:** Attitude toward marine environment and sustainability of seafood by socio-demographic characteristics

Variables	Mean	SD
Overall	4.54	0.65
Gender		
Male	4.50	0.69
Female	4.56	0.61
Age^*^		
18–34	4.43	0.68
35–64	4.53	0.67
65 and older	4.75	0.44
Ethnicity^*^		
European and North American	4.59	0.62
Other	4.44	0.69
Education		
Less and equal to high school	4.47	0.74
College, CEGEP, non-bachelor certificate/diploma	4.52	0.60
University	4.50	0.72
Above Bachelor or Graduate degree	4.65	0.48
Household Income		
< $40,000	4.51	0.54
$40,000 to $99,999	4.53	0.66
$100,000 and over	4.55	0.68
Children in the Household		
Yes	4.48	0.73
No	4.58	0.59

^*^p < .05

Overall, responding consumers presented a high level of awareness of the importance of marine environment protection and harvesting seafood sustainably with a mean score of 4.54. The perceived importance varied significantly by age and ethnicity. The elderly (65 years and older) and those indicating a European or North American background reported a higher level of perceived importance for protecting the marine environment and sustainability of seafood compared to other categories. However, there was no significant difference in the perceived importance by gender, education, household income, and children in the household.

### Understanding the meaning of seafood sustainability

To assess the consumers’ understanding of seafood sustainability, respondents were asked to choose what a sustainable seafood product indicates to them. About 36% of respondents fully understood “sustainable”—choosing all the correct answers. More than 40% of respondents chose at least one correct answer, but they also confused “sustainable” with “fresh, raw, or over the counter,” “organic written on the label,” or “local written on the label.” More than 23% did not understand the meaning at all, choosing all the incorrect answers.

Table 3 presents results of bivariate analysis of understanding seafood sustainability by socio-demographic characteristics. There were significant differences in understanding the meaning of seafood sustainability by gender, ethnicity, and household income. A higher percentage of males than females were found in categories “full understanding” and “no understanding.” Females were more likely than males to have a “partial understanding” of seafood sustainability, but together with those who had a full understanding, females had a better understanding compared to males. Among age groups, the elderly had the highest percent of “no understanding,” while the 35–64 age group had the highest percent of “full understanding.” The 18–34 age group had the lowest percent of “no understanding” but had the highest percent of partial understanding (54%). Having European or North American background and having household income of $100,000 or more were associated with “full understanding” about the meaning of seafood sustainability. There were slight differences in understanding seafood sustainability by education and children in the household, but the differences were not statistically significant.

**Table 3:** Understanding seafood sustainability by socio-demographic characteristics

Variables	Not at all	Partial understanding	Full understanding
Overall	23.2	41.1	35.8
Gender^**^			
Male	28.6	31.8	39.6
Female	19.2	48.3	32.5
Age^***^			
18–34	15.2	53.5	31.3
35–64	24.4	34.5	41.1
65 and older	35.7	42.9	21.4
Ethnicity^**^			
European and North American	21.5	37.1	41.4
Other	26.5	50.4	23.1
Education			
Less and equal to high school	30.4	32.1	37.5
College, CEGEP, non-bachelor certificate/diploma	30.2	37.5	32.3
University	20.3	43.0	36.7
Above Bachelor or Graduate degree	14.1	48.7	37.2
Household Income^*^			
< $40,000	28.6	37.5	33.9
$40,000 to $99,999	25.9	45.6	28.6
$100,000 and over	15.8	38.8	45.3
Children in the Household			
Yes	22.1	40.2	37.7
No	23.8	41.6	34.6

^*^p < .05, ^**^ *p* < .01, ^***^ *p* < .001

### Awareness of sustainable certification or ecolabel^2^

Table 4 shows how much respondents recognize specific sustainable seafood certifications or ecolabels found in Canadian food retail venues and whether they look for the certifications or labels when shopping.^3^ The respondents’ level of recognition and understanding varied across the labels. Depending on the ecolabel, 56.1% to 93% of respondents were not able to recognize any particular ecolabel. Among the nine labels, the most well-recognized ecolabel was the Dolphin Safe (43.9%), followed by Ocean Wise (41.3%), Responsible Fishing Scheme (39.7%), Aquaculture Stewardship Council (ASC) (31.3%), and Marine Stewardship Council (MSC) (31.3%). Over 20% of respondents recognized Best Aquaculture Practices (BAP), SeaChoice (pocket guide), and Friend of the Sea. Only 7% of respondents recognized the ThisFish traceability tool.

**Table 4:** Recognition, understanding, and looking for seafood with sustainable certifications and ecolabels (percent)

	(1) Recognizing ecolabels				(2) Looking for label when shopping	
	Yes			No	Yes	No
	Recognize only	Recognize and understand	Total			
Dolphin Safe	24.9	19	43.9	56.1	25.9	74.1
Ocean Wise	28.2	13.1	41.3	58.7	25.6	74.4
Responsible Fishing Scheme	31.3	8.4	39.7	60.3	25.3	74.7
Aquaculture Stewardship Council (ASC)	22.9	8.4	31.3	68.7	12.8	87.2
Marine Stewardship Council (MSC)	22.1	9.2	31.3	68.7	13.7	86.3
Best Aquaculture Practices (BAP)	13.7	8.9	22.6	77.4	8.9	91.1
SeaChoice (pocket guide)	14.8	7.5	22.3	77.7	4.2	95.8
Friend of the Sea	14.8	7.5	22.3	77.7	8	92
ThisFish traceability tool	3.6	3.4	7	93	2.4	97.6

This table presents respondents’ answers for two separate questions regarding each sustainable certification/ecolabel: (a) Whether they recognize only, recognize and understand, or do not recognize each label (*n* = 358); and (b) whether they look for when purchasing fish or seafood (*n* = 336)

Regarding respondents’ understanding of the label, Dolphin Safe (19%) was the most well-understood label, followed by Ocean Wise (13.1%), MSC (9.2%), BAP (8.9%), ASC (8.4%), Responsible Fishing Scheme (8.42%), SeaChoice (7.5%), Friend of the Sea (7.5%), and ThisFish traceability tool (3.4%). As shown in Table 4, many respondents who reported they recognized the label did not understand what each label signified. For example, about 40% of respondents reported that they recognized the label of Responsible Fishing Scheme, while only 21.2% of those who recognized the label (8.4% of respondents as indicated in the Table 4) reported that they understand what the label signified, and about 79% simply recognized the label without any understanding. Similar patterns were found with other labels such as Ocean Wise where 41.3% of respondents recognized the label but only 13.1% (31.7% of those who recognized the label) reported that they understood what the label signified. When shopping for seafood, Dolphin Friendly, Ocean Wise, and Responsible Fishing Scheme were the three ecolabels that the respondents looked for the most, with more than 25% reporting positively for each of them.

The main source of information for respondents about ecolabeled or sustainable seafood was online resources and/or mobile applications (44.8%), followed by detailed product labels (43.4%), television documentaries, lifestyle television or celebrity chefs (36.6%), word of mouth (29%), newspaper and magazine articles (27.3%), in-store help/consulting (19.2%), and others (16.3%).

### Purchasing seafood with sustainable certification or ecolabel

Respondents were asked to rank in importance the factors that play in their purchasing decision for seafood. Having a sustainability/ecolabel (52%) was ranked sixth following “flavor” (91%), “freshness” (86.7%), “contaminants” (75.9%), “health benefits” (71.1%), and “price” (62.2%).

With respect to actually purchasing ecolabeled seafood in the supermarket when available, 19.2% of respondents reported that they purchased “always or most of the time,” 51.9% purchased “often or sometimes,” and 28.9% responded “rarely or never.” While dining in restaurants, only 5.9% of respondents reported that they ordered ecolabeled seafood “always or most of time,” 38% reported “often or sometimes,” and 56.1% indicated “never or rarely” when available (see Table 4). Purchasing behaviors of sustainable seafood were significantly different by gender, age, attitude toward marine environment and sustainability of seafood, and the level of understanding. Males and the elderly were more likely than their counterparts to “never or rarely” purchase the sustainable seafood. It is important to note that those with higher level of perceived importance for the marine environment and sustainability of seafood, and those with a higher level of understanding, reported to more frequently purchase sustainable seafood in the supermarket and to order it in the restaurant. About 60% of those who did not understand the meaning of seafood sustainability “never or rarely” purchased sustainable seafood in the supermarket, compared to 26.5% with “partial understanding” and 14.4% with “full understanding.” With respect to ordering sustainable seafood in a restaurant, almost three quarters of respondents who did not understand the meaning at all ordered “never or rarely,” compared to 52.8% of partial understanding and 49% of full understanding.

Barriers.

Table 5 shows the key barriers that respondents experienced in purchasing sustainable/ecolabeled seafood. Lack of understanding of seafood certification products and ecolabels on seafood (66.5%) was identified as the top barrier, followed by the lack of information available about sustainable seafood products (55.6%). Forty-three percent of respondents mentioned “price” as an important obstacle to purchase ecolabeled seafood. The absence of in-store assistance and/or resources about sustainable options and the difficulty in locating certified products in-store were also identified as important barriers (41% and 23.5%, respectively). Interestingly, more than a third of respondents mentioned that the existence of many different ecolabels on seafood products was a challenge for purchasing ecolabeled seafood (Table 6)(ADD QUERY HRE).

**Table 5:** Purchasing behaviors of sustainable seafood by socio-demographic characteristics, attitude toward marine environment and sustainability of seafood and understanding seafood sustainability (percent)

Variables	Purchasing in the Supermarket			Ordering in the Restaurant		
	Never/rarely	Sometimes/Often	Most of the time/always	Never/rarely	Sometimes/often	Most of the time/always
Overall	28.9	51.9	19.2	56.1	38.0	5.9
Gender^a*,b+^						
Male	37.1	45.5	17.4	62.4	35.0	2.6
Female	23.2	56.8	20.0	51.8	40.0	8.2
Age^a**^						
18–34	26.4	56.3	17.2	49.4	42.0	8.6
35–64	23.3	56.3	20.5	55.3	40.3	4.4
65 and older	53.1	32.7	14.3	69.8	23.3	7.0
Ethnicity						
European and North American	28.8	49.1	22.2	57.1	36.1	6.8
Other	30.4	55.9	13.7	54.8	40.9	4.3
Education						
Less and equal to high school	36.2	51.1	12.8	55.0	42.5	2.5
College, CEGEP, non-bachelor certificate/diploma	27.1	58.8	14.1	61.3	34.7	4.0
University	25.0	51.8	23.2	55.1	36.4	8.4
Above bachelor or graduate degree	32.4	44.6	23.0	52.3	41.5	6.2
Household income						
< $40,000	28.3	63.0	8.7	56.1	41.5	2.4
$40,000 to $99,999	28.0	53.0	18.9	53.0	39.1	7.8
$100,000 and over	27.2	48.8	24.0	56.4	37.6	6.0
Children in the household						
Yes	26.2	52.3	21.5	57.7	40.2	2.1
No	30.0	51.7	18.4	55.4	36.6	8.1
Attitude toward Marine Environment and Sustainability of Seafood^a***, b*^	4.37 (0.86)	4.53 (0.58)	4.82 (0.35)	4.48 (0.67)	4.67 (0.46)	4.63 (0.65)
Understanding Seafood Sustainability^a***, b*^						
Not at all	58.8	36.8	4.4	73.4	23.4	3.1
Partial understanding	26.5	53.0	20.5	52.8	38.6	8.7
Full understanding	14.4	59.3	26.3	49.0	46.9	4.2

^a^: *p < .05, **p < .01, ***p < .001

^b^: *p < .05; ^+^*p* < .1

^c^: Mean (standard deviation)

**Table 6:** Key barriers to purchasing sustainable/ecolabel seafood (percent)

Barriers	Yes	No
Limited understanding of seafood certification products and labels	66.5	33.5
Limited information on product sustainability	55.6	44.4
The price of certified sustainable seafood products	43.0	57.0
Lack of in-store help and/or resources about sustainable options	41.0	59.0
Too many certification labels	34.7	65.3
Difficulty in locating certified products in-store	23.5	76.5
Other	9.2	90.8

*n* = 349. This is a multiple response measurement. Thus, the total percent of all the response categories is not 100

## Discussion and conclusion

We have argued that sustainable consumption is an integral component of ecological citizenship. In order to increase sustainable and responsible consumption, it is important to understand the consumer/citizens’ awareness of the environmental impacts of the products they purchase, their purchasing behavior, and the perceived barriers to the uptake of these products in the market place. While some survey research has explored consumer engagement with sustainable/ecolabeled seafood in several European contexts and in the USA, Canada remains relatively understudied. As the first of its kind in the Canadian context, our survey research provided an overall picture of the Canadian consumers’ awareness about the importance of the marine environment and sustainability of seafood, understanding of the meaning of seafood sustainability and sustainable certifications, their purchasing behavior with respect to sustainable seafood products, and the perceived barriers to the uptake of these products.

One of our major findings highlights that Canadian consumers were well aware of the importance of marine environment protection and the use of sustainable seafood harvesting methods, and this awareness was considerably higher than the results of a Swedish study that asked similar questions (Jonell et al. 2016). In contrast, the majority of Canadian consumers either did not understand the meaning of “sustainability” of seafood at all or often confused it with “local,” “fresh,” “raw,” and “organic.” Moreover, most consumers did not understand what the ecolabels found in the Canadian marketplace meant, though some seafood ecolabels were fairly well recognized. There is, then, a considerable gap between consumers’ awareness of the importance of marine environment protection and sustainability of seafood and the level of understanding of the meaning of seafood sustainability, which would permit more sustainable consumption practices. This suggests that the great majority of seafood consumers lack the knowledge base to make effective purchasing decisions that would support sustainably harvested seafood. This result is in line with European studies (Jonell et al. 2016; Pieniak et al. 2013) and a comparative survey in the USA and UK (Guiterrez and Thornton 2014) and one in Australia (Lawley et al., 2019:2346). Such low level of understanding of the meaning of sustainability and the sustainable seafood certifications/ecolabels may explain the low ranking of “ecolabeled seafood” as a determinant of purchasing decision for seafood in our study. It only ranked 6^th^ after flavor, freshness, contaminants, health benefits, and price. It might also reflect why almost a third (29%) of responding consumers “rarely” or “never” bought ecolabeled seafood when available, compared to 16% in the Swedish study (Jonell et al. 2016). The absence of a common standard of sustainability among the various ecolabel organizations is likely an obstacle here and needs to be tackled in future, an issue we address below.

The Canadian consumers’ levels of awareness of the importance and understanding of seafood sustainability, and their purchasing behavior of sustainable seafood were different by some socio-demographic characteristics. Awareness of the importance of marine environment protection and harvesting seafood sustainably were significantly higher among the elderly and those having European or North American background. Meanwhile, there was no significant gender difference, which was not consistent with the findings of large European studies on a wide range of food products (Grunert et al. 2014) and on ecolabeled fish (Brecard et al. 2009) which found women to have a higher concern with sustainability. Regarding *understanding* the meaning of seafood sustainability, females, younger consumers, particularly those in the 35–64 age group, those having European or North American background, and those in the highest income category had a better understanding about seafood sustainability compared to their counterparts in our survey. Interestingly, significant differences in understanding were not found with respect to education level, in contrast to a European study examining attitudes towards sustainability with respect to food (Gunert et al. 2013).

With respect to *purchasing* behavior, our study found that females were more likely to prefer or purchase sustainable seafood products, a finding also reported in studies conducted in some European countries (Brecard et al. 2009; Johnston et al., 2001). Younger consumers were more likely to purchase sustainable seafood in our study as was the case in a French survey (Salladarré et al. 2010) and a survey conducted in several European countries (Brecard et al. 2009). It is noteworthy that those in our study who indicated the highest level of concern for the marine environment and sustainable harvesting, those 65 and older, were the least likely to purchase ecolabeled seafood “most of the time/always.” A study in Mexico (Pérez-Ramirez et al. 2015) and one in Europe (Grunert et al. 2014:187) also found older consumers the least likely purchasers of such products as well. A better understanding of these contradictory results will have to await further research on the age factor in consumer decision-making. In contrast to studies conducted in Korea and China, respectively (Kim and Lee 2018; Wang and Somogyi 2019), our study did not find a significant relationship between income and likelihood of purchasing sustainable seafood. This would suggest that income might not be a barrier to the increased purchasing of such products in the Canadian context.

It should be mentioned that environmental literacy does not automatically translate into sustainable consumption practices. Consumption in the market place entails multi-layered meanings (Seyfang 2005:297) involving such considerations as identity, loyalty to one’s ethnic group, self-expression, convenience, affordability, and so on and, in the case of seafood, freshness understandably factors in for most people. However, there is some empirical evidence that understanding of seafood sustainability and ecolabels and/or awareness of the negative environmental effects of seafood production *are* good predictors of preference for ecolabeled seafood (Brécard et al. 2009; Jonell et al. 2016; Pérez-Ramírez et al. 2015). Our study findings also showed that awareness of the importance of marine environment protection and sustainable seafood production as well as understanding the meaning of seafood sustainability had strong significant relationships with purchasing sustainable seafood, in line with the few other studies that have examined this (Almeida et al. 2015; Jonell et al. 2016; Lawley et al. 2019).

The importance of awareness and understanding of seafood sustainability in general and understanding of seafood certification or ecolabels for the increased purchasing of sustainable seafood product was echoed in the assessment of consumers’ perceived *barriers* to purchasing sustainable seafood product. Our study findings highlight that the limited understanding of seafood certification products and labels as well as product sustainability were the most prominent barriers. Given these results, it seems fair to ask who needs to play a role in educating the consuming public about the broader issues of seafood sustainability and the more specific matter of what ecolabels actually signify? Here, we would suggest that the obvious candidates are the ecolabel certification programs themselves, the food retail sector, and the state.

Several prominent seafood certification programs have been in existence for many years yet our study clearly indicates that Canadian consumers have little in the way of even a basic understanding of what their labels actually signify. We believe that ecolabel programs have to do a much more effective job in communicating the meaning and value of sustainably certified seafood to the consuming public. In addition, the perceived lack of in-store assistance and resources to guide sustainable seafood purchasing points to the urgent need for supermarket chain store companies to step up and provide their customers with the information they need at point-of-purchase to choose sustainable seafood options. This need was also noted in a Swedish study (Jonell et al. 2014:10). The existing working relationship between some major retailers and certification organizations suggest that this could be a cooperative effort to improve in-store messaging. In addition, “too many certification labels” was indicated as an important barrier in our study and this reality has been noted by other studies (e.g., Wessels et al. 1999; Horne 2009) as a source of confusion among consumers wishing to make ecologically sensitive seafood purchases. Collaboration of certification organizations directed towards the goal of reducing the number of labels would help reduce consumer confusion and simplify the task of “doing the right thing” with positive benefits for sustainability. However, overcoming different criteria around what constitutes sustainable seafood is likely to be a difficult obstacle in the way of such collaboration, and here is perhaps where governments and intergovernmental organizations could play a role in establishing an agreeable minimal standard of sustainability with respect to ecolabel programs and making this known to the consuming public.

With respect to this role the state then, some have argued it needs to be more active in shaping consumer choices through education to shift social values and regulations to force corporations and citizens to make more environmentally friendly choices (de Bakker and Davegos 2012:890; Seyfang 2005:303) and strengthen the efficacy of existing ecolabel programs (Horne 2009:180). However, in the seafood industry, public and private governance around sustainability has emerged in rather complex ways. In the private sphere, there has been growing pressure from environmental NGO’s and from powerful corporate buyers, often responding to pressure from these NGOs, to bring more sustainable practices to the seafood sector. Historically, the growing influence of the Marine Stewardship Council, a private governance body, has served as a disrupter and has spurred the intergovernmental Food and Agriculture Organization (FAO), under pressure from European Nordic countries themselves concerned with the MSC’s growing influence, to elaborate “globally applicable minimum criteria” to guide the development of seafood ecolabeling initiatives, essentially transnational governance norms (Foley and Havice, 2016:26). Beyond this response, and related to the MSC’s rising influence, has been a pushback from private sector seafood interests and some governments—notably in Iceland, Japan, and the state of Alaska—through the establishment of what Foley and Havice have called territorial eco-certification regimes. These are seen as shoring up “the territorial, often national, industry identities and features of place-specific production and state-based regulatory regimes ….” (2016: 31). These represented an effort to gain control over wild-caught fisheries certification that was viewed as increasingly monopolized by the MSC, a private organization which was beyond national jurisdiction (Gullbrandsen, 2012). Nevertheless, rather than a rejection of external pressures for more sustainable practices in the seafood industry, these territorial-based public regimes have continued to embrace what has been termed “transnational governance norms” around sustainable seafood, and notably those elaborated by the FAO (Foley and Havice, 2016: 31). Gullbrandsen (2012) has argued that because MSC standards effectively emulate those principles around seafood sustainability elaborated by the FAO, governments have come to view MSC’s certification program as a useful supplement to domestic management regimes and have increasingly supported the MSC’s efforts. In the seafood sector, then, private–public governance interactions, at least to some degree, have been to the benefit of enhancing sustainability. Returning to the issue of the proliferation of seafood ecolabels and the confusion this is causing the consuming public, one role governments could play is to use the voluntary governance norms already elaborated by the FAO as a *mandatory* guide to what minimal meaningful and verifiable standards any sustainable seafood ecolabel would *have* to comply with in their national jurisdictions. This is a view others have advocated as well (see Foley and Havice 2016:27; Food and Water Watch, 2010:14).

While there is evidence of a more active public governance in some national and international contexts with regard to protecting wild-caught fisheries—it must be recognized that there is considerable governmental backsliding as well. This includes regions where more progressive public policies might be expected, such as the European Union, which has turned a blind eye to the damaging and destructive fishing practices of its fishing fleets in the Indian Ocean, for example, and failed to *effectively* protect Mediterranean designated marine protected areas (see Harvey 2019; Holland 2021; McVeigh 2021). Therefore, we argue that the place of *private* governance mechanisms such as seafood ecolabels and the engagement of the consuming public continues to have considerable relevance. This view is supported by decisions of some global food retailers and suppliers (Walmart, Tesco, Carrefour, McDonalds and Sysco) to partner with the MSC to source at least some of their supply (MSC n.d.; Sysco 2020). With respect to the consuming public, the issue of how to educate and engage the end buyer cannot be overlooked not only because the role consumers can play in supporting the decisions that more progressive food retailers and suppliers have made (Barclay and Miller 2018), but also in pushing the laggards in the private retail sector to up their game. Educated consumers could also be an important force pushing for environmentally responsible fishing policies from governments that hitherto have been under the sway of private sector interests opposed to change.

To return to the theoretical concept of ecological citizenship introduced in the beginning of this report, Seyfang (2005) has argued that we need “to identify the conditions under which ecological citizenship is developed and how it might be nurtured.” Wolf et al. (2005:505) have argued that ecological citizenship “calls at least for an acknowledgement of the relative environmental impact of a citizen and at best for efforts to reduce it.” Our respondents overwhelmingly indicated that they favored an ethical approach to the marine environment and seafood harvesting, but our findings indicate some of the key barriers that hinder consumer/citizens from actualizing environmentally responsible consumption of seafood. Concerted efforts to address these barriers could have, we believe, very salutary effects in the seafood sector and need not preclude a more robust regulatory regime from government to ensure that ecolabels deliver what they promise for the end consumer.

While we believe our study makes an original contribution to a little-known subject of consumer interface with sustainable seafood in Canada, there is a limitation on the generalizability of our findings to the whole Canadian context. Due to the unfeasibility of achieving a list of all seafood consumers in Canada, we employed a nonprobability sampling. However, our site selections helped increase the fit between our sample characteristics and those of the Ontario provincial population. In addition, this study is focused on the Toronto Metropolitan Area and environs. While this is the largest metropolitan area in Canada, the country has some significant regional variations. Future research will need to tap into other regions of the country to determine the extent to which geography makes a difference.

## Footnotes

1 However, with respect to the efforts to curb IUU fishing in the tuna fishery, the International Seafood Sustainability Foundation reported in March 2021 “significant gaps in the implementation of proposed counter-measures by five tuna regional fishery management organizations (RFMOs)” (Oirere 2021).

2 We provided the name of nine sustainable seafood certification and ecolabels found in Canadian food retail stores, incorporating the graphics of each logo in its original colors just as it would appear on the product.

3 We have not examined the socio-demographic influences on recognition and understanding of each ecolabel as the study was focused on sustainable seafood products more generally and not on the particular seafood ecolabels found in the Canadian market. Moreover, the sample size did not allow for meaningful statistical analysis to be carried out for all the labels that were found in the retail sites studied.
